# Interrelationship between silicon, aluminum, and elements associated with tissue metabolism and degenerative processes in degenerated human intervertebral disc tissue

**DOI:** 10.1007/s11356-017-9588-y

**Published:** 2017-07-07

**Authors:** Anetta Zioła-Frankowska, Łukasz Kubaszewski, Mikołaj Dąbrowski, Marcin Frankowski

**Affiliations:** 1 Department of Analytical Chemistry, Faculty of Chemistry, Adam Mickiewicz University in Poznań, Umultowska 89b, 61-614 Poznan, Poland; 2 Department of Spondyloortopaedics and Biomechanics of the Spine, W. Dega University Hospital, Poznan University of Medical Sciences, 28 Czerwca 1956 135/147, 61-545 Poznan, Poland; 3 Department of Water and Soil Analysis, Faculty of Chemistry, Adam Mickiewicz University in Poznań, Umultowska 89b, 61-614 Poznan, Poland

**Keywords:** Silicon, Aluminum, Intervertebral disc tissue, Spine, Si-Al correlation

## Abstract

There is a growing body of evidence concerning the significant role of silicon in development and composition of both connective and bone tissue. Bio-essential silicon shows strong chemical and biological affinity to aluminum, which is toxic and biologically inessential element. The presence of silicon was confirmed in a variety of tissues; however, it has never been examined in intervertebral disc tissue, neither in healthy nor in degenerated one. In this paper, for the first time in the literature, we present the content of silicon in the degenerated intervertebral disc tissue. We also compared the results of silicon analysis with aluminum values in degenerated intervertebral disc tissue in humans. We used chemometric methods to find correlations and similarities between silicon, aluminum, and elements associated with tissue metabolism (Mg) and degenerative processes (Zn and Cu). The presence of silicon was confirmed in all 30 samples harvested from 22 patients operated on due to degenerative changes. Its concentration was within the range of 5.37–12.8 μg g^−1^ d.w., with the mean concentration of 7.82 μg g^−1^ d.w. The analysis showed significant correlation between Si and both Al and Mg and weak or negative correlation with Zn and Cu, where the latter was probably the result of degenerative processes. Although silicon is considered essential in glycosaminoglycan and collagen synthesis in connective tissue, it did not show any correlation nor similarities with elements reflecting changes associated with the degenerative process of the intervertebral disc. Silicon showed significant correlation with aluminum, similar to those observed in other human tissues.

## Introduction

Until the 1970s, silicon was considered as a biologically insignificant element. Even now, some authors tend to recognize silicon as a non-essential microelement. The first studies that hypothesized its significance in bone formation and glycosaminoglycan composition were published in the 1970s (Carlise 1970). Further analyses confirmed its important role in bone and connective tissue, and its influence on mechanical properties and endurance of the tissues (Nielsen and Sandstead 1974; Carlisle 1981; Lugowski et al. 1998; Jugdaohsingh 2007; Kim et al. 2014). Based on the abundance in the human body, we may distinguish three groups of chemical elements, depending on their concentration in tissues: (1) major dietary elements such as calcium, phosphorus, potassium, sulfur, sodium, chlorine, and magnesium; (2) minor dietary or trace elements such as iron, cobalt, copper, zinc, manganese, molybdenum, iodine, bromine, and selenium; and (3) others, considered as “ultratrace” elements (due to very low concentration and not clear role)—boron and chromium. The elements such as arsenic and silicon, due to lack of sufficient studies, cannot be attributed to any of the groups. Other classification divides elements into three major groups with respect to their biological role: essential, potentially essential, or not essential for the organism (Kabata-Pendias and Mukherjee 2007). The essentiality of elements is to some degree connected with their tissue concentration in dry mass, reflecting the number of biochemical processes in which they take part. The metabolically significant elements are usually within concentration ranges expressed in mg/kg, e.g., magnesium, zinc, or copper (Nowakowski et al. 2015; Kubaszewski et al. 2014). Potentially significant elements are within one order of magnitude lower concentration ranges, i.e., μg kg^−1^ d.w. Ni, Co, Mo with the available analytical techniques they may not be detected in every sample (Kubaszewski et al. 2014). The silicon is considered to be a transition element. In the periodic table, it is placed between nonmetals and metals. The proximity of silicon to carbon, some similarities to this organic compounds backbone, as well as the abundance in the environment, may indirectly suggest silicon significance in biochemistry. Indeed, skeletons of primitive animals, bacteria, and plants (diatoms, radiolaria, siliceous sponges) are built of silicon (Exley 2009a). Its role in humans is ultimately not defined (Martin 2013). The studies show that it plays a crucial role in collagen-rich tissues integrity (nail, skin, hair), collagen synthesis, bone tissue development and regeneration, as well as in the reduction of aluminum accumulation, which reflects the risk of Alzheimer’s disease (Schwarz and Milne 1972; Exley et al. 2006; Rodella et al. 2014; Jugdaohsingh et al. 2015). Silicon is a substantial element of collagen and glycosaminoglycan-rich tissues (Reffitt et al. 2003; Spector et al. 2005; Pruksa et al. 2014). One of such tissues is the intervertebral disc (IVD). In IVD, both collagen dysfunction and glycosaminoglycan decomposition are considered a direct cause of the degeneration process, with loss of the biomechanical function—clinically symptomatic back pain, resulting in substantial costs to the health care system (Nowakowski et al. 2015). To our knowledge, there have been no analyses or published papers on silicon concentration in degenerated IVD. Also, silicon in the form of silicic acid is regarded as a natural antagonist of aluminum toxicity in biota and in humans. It reduces aluminum uptake across the gut and simplifies the excretion of systemic aluminum via kidneys (Exley 2009a; Davenward et al. 2013). Moreover, the studies presented an evidence that long-term drinking of silicon-rich mineral water significantly reduces the body burden of Al (Exley et al. 2006; Davenward et al. 2013). Silicon is also important for the immune system and prevents arteriosclerosis. However, officially, it is still not considered as an essential nutrient for humans (Martin 2013).

In spite of the abovementioned facts, our knowledge on silicon concentration and distribution in human tissues is not even satisfactory. It is considered as the third most abundant microelement (1–10 mg kg^−1^) in humans. However, this assumption is based on data derived from “insignificant” tissues such as the hair, nails, and epidermis. In the literature, we can also find some results of silicon analysis in the serum and urine, which are discussed in terms of Si intake, accumulation, and excretion characteristics (Jugdaohsingh 2007). To our knowledge, so far, human mechanical tissues have not been analyzed for Si concentration and Si-Al relations.

The aim of this research was to determine the concentration of Si in degenerated IVD in humans, by ICP-OES analytical technique, and check the correlations between Si and Al, Cu, Zn, and Mg, found in the analyzed tissues, by statistical methods.

## Materials and methods

A total of 30 samples of degenerated IVD were harvested from 22 patients undergoing surgical discectomy or spinal fusion due to degenerative disc disease. The samples were obtained from the cervical spine (12 samples from 6 patients) and from the lumbar spine (18 samples from 16 patients). The pre-operative magnetic resonance images, taken to evaluate degeneration status of the operated disc, were scored according to the Pfirrmann classification (Pfirrmann et al. 2001). The use of tissues for this study was approved by the appropriate bioethics committee, and the written consent was obtained from all patients. The permission was given by the Bioethics Committee of Institute of Rheumatology, Warsaw, on May 31, 2012, and Bioethics Committee of University of Medical Sciences, Poznań, (No.406/13). The average age of patients at the time of operation was 47.6 (range 28–64 years). The information on demography, health status, and occupational exposure to heavy metals was collected from all patients. The patients involved in the study were not exposed to heavy metal pollution.

The degenerated IVD samples were frozen. Prior to the analysis, they were freeze-dried using a Lyovac lyophilizer GT2e (Steris, Germany), for 24 h. The samples were weighed after drying and placed in Teflon vessels. The supra-pure nitric acid (V) (Merck, Germany) was added to obtain a maximum dilution factor of 10. The amounts of dry samples ranged from 0.2 to 0.6 g, and the amounts of acid were from 2.0 to 6.0 ml. Then, samples with acid were left to stand overnight to slow mineralization. After this step, samples were mineralized in a microwave oven (Mars Xpress 5, CEM USA), based on the modified 3051 EPA method (Zioła-Frankowska et al. 2015, 2016). In the case of Si, 200 μL of 1% supra-pure hydrofluoric acid (Merck, Germany) was added to the extracts of IVD tissues to prevent the precipitation of silicon in an acid medium. The concentration of Si was determined using a Shimadzu ICPE 9820 ICP-OES spectrometer (Shimadzu, Japan). The optimized operating conditions for the analysis of Si in IVD samples are presented in a table (Table 1).

**Table 1:** Operating conditions and accessories employed in ICP-OES spectrometer (Shimadzu ICPE-9820) for analysis of intervertebral discs samples

Parameter and accessories		Value
Radio frequency power generator		1.2 kW
Gas type		Argon
Plasma gas flow rate		10.0 L min^−1^
Auxiliary gas flow rate		0.6 L min^−1^
Nebulization gas flow rate		0.7 L min^−1^
Plasma view		Vertical torch; axial view
Selected emission lines (atomic)		251.611 (BEC = 0.084)
		212.412 (BEC = 0.188)
Torch		Mini-torch (quartz)
Nebulizer		Burgener NX-175
Chamber		Cyclone (glass)
Drain		Gravity fed
Injector tube		Quartz (1.2 mm i.d.)
Background correction		2-points
Number of replicates		3
Exposure time		15 s
Peristaltic pump	Solvent rinse	15 s
	Sample rinse	15 s
	Sample uptake rate	1 ml min^−1^
Spectrometer	Echelle optics	Range of wavelength:167 to 800 nm
	Resolution	≤0.005 nm at 200 nm
	Atmospheric removal system	Rotary vacuum pump ≤10 Pa
Device	CCD (charge coupled device) detector	
	Pixel number	1024 × 1024 pixels (1 in.)
	Pixel size	20 μm × 20 μm
	Cooling control	Peltier device

The concentration of Al and Cu was determined by a Shimadzu AAS 7000 spectrometer (Shimadzu, Japan) with graphite furnace atomization (GF-AAS). The concentration of Mg and Zn was determined with the use of the Shimadzu AAS 7000 with flame atomization (F-AAS). The optimized parameters for determination of metals have been presented in the study (Kubaszewski et al. 2014). The concentrations of the other metals, obtained by Kubaszewski et al. (2014) are presented in Table 2.

**Table 2:** Concentration of Al, Cu, Mg, and Zn in intervertebral disc (Kubaszewski et al. 2014)

Element	AM	S.D.	Range
	[μg g^−1^ d.w.]		
Al	0.664	0.289	0.166–1.271
Cu	3.41	4.045	0.97–23.64
Mg	800.1	525.5	182.6–2132
Zn	39.60	35.95	10.56–184.5

*AM* arithmetic mean, *S*.*D*. standard deviation

To estimate the correlation of silicon and the selected metals, the statistical analysis was performed with Statistica v. 7.0 (StatSoft, Poland). In the standardization process, all values of selected variables were replaced by standardized values, which were computed as follows: Std. score = (raw score − mean)/Std. deviation. The Spearman rank-order correlation analysis was performed to determine correlation between Si and the other elements (Al, Cu, Mg, and Zn) and epidemiological data (age, sex, degeneration based on Pfirrmann score, area of degeneration: cervical or lumbar spine). Moreover, chemometric analysis of the analyzed elements was performed. After autoscaling, the similarity analysis was performed with graphical methods. Tangent distances (*d* ^T^) were calculated and relationships between the elements were analyzed using a dendrogram.

## Results

The results of such research could reveal the role of silicon in IVD, as well as promote new studies on the pathogenesis and the possible treatment. In this study, we present the concentration of silicon in degenerated human IVD. Furthermore, we try to find relationships between Si and metabolically related elements such as Mg, Zn, and Cu. As silicon presents both ex vivo and in vivo high affinity to toxic aluminum, we also included this element in the study. The measured concentrations of silicon were from 5.37 to12.8, with the mean of 7.82 and SD of 1.76 (all in μg g^−1^ d.w.). Moreover, we analyzed the results according to the age and gender of patients included in the study. In the group of women, the lowest obtained concentration of Si amounted to 5.37 μg g^−1^ d.w. (42 years old female), and the highest was 10.7 μg g^−1^ d.w. (28 years old female). In men, the lowest determined concentration of Si amounted to 5.72 μg g^−1^ d.w. (38 years old male), and the highest was 12.8 μg g^−1^ d.w. (61 years old male). We observed that in females, the highest concentration of Si occurred in a patient under 40 years old, and in males—in a patient who was over 40 years old.

Based on the results of the statistical analysis, it was observed that Si showed significant positive correlation with both Mg and Al, no correlation was found for Si and Zn, and a significant negative correlation was found for Si and Cu. Also, there was no correlation between Si and age, sex, and degeneration score based on the Pfirrmann classification (Pfirrmann et al. 2001). The correlations between the analyzed elements in the intervertebral disc were described by the Spearman rank correlation (Table 3).

**Table 3:** Spearman’s rank correlation order of the elements measured in intervertebral disc samples

Spearman	Al	Si	Cu	Mg	Zn
Al	x				
Si	0.47*	x			
Cu	−0.25	−0.50*	x		
Mg	0.50*	0.46*	−0.32	x	
Zn	0.27	0.09	−0.08	0.75*	x

*Statistically significant; *p* value <0.01

Statistically significant higher content of Si was observed in men. The cervical disc in comparison to lumbar disc was characterized by statistically significant lower Si content. There was no statistical difference in Si content between patients who had undergone a surgery with implants and those who had not had such surgery. The results of statistical analysis are presented in Table 4.

**Table 4:** Silicon concentration in intervertebral disc (in μg g^−1^ d.w.) according to the selected factors

Factors		*N*	AM ± S.D. [μg g^−1^]	Med. (QL-QU) [μg g^−1^]	Mann-Whitney *U* test
Gender	Men	18	8.3 ± 1.6	8.3 (7.2–8.6)	0.046
	Women	12	7.1 ± 1.8	6.5 (5.6–8.3)	
Implants	No	21	8 ± 1.9	8.3 (6.6–8.8)	0.46
	Yes	9	7.4 ± 1.5	7.4 (6.5–8.3)	
Intervertebral disc from	Lumbar	18	8.5 ± 1.8	8.4 (7.2–9.7)	0.016
	Cervical	12	6.8 ± 1.2	6.5 (5.7–8.1)	

*N* number of samples, *Med*. median, *QL* lower quartile, *QU* upper quartile

Based on the results of the Spearman correlation (Table 5), it was found that Si concentration significantly increased with concentrations of Al and Mg and decreased with Cu concentration. We observed gender differences in the correlation between Si and Mg—a statistically significant increase in Si concentration in the intervertebral disc with the increase in Mg concentration was noticed only in female patients. Statistically significant positive correlations between Si and Pb, Zn, and age were found only in patients who had undergone a surgery with implants.

**Table 5:** Spearman rank correlation between Si, the other analyzed metals, and clinical factors (gender, implants, type of spine, patient’s age, and Pfirrmann et al. (2001) score)

		Sex		Implants		Intervertebral disc from	
Si		Men	Women	No	Yes	Lumbar	Cervical
Al	0.47*	0.35	0.48	0.53*	0.77*	0.59*	0.48
Cu	−0.40*	0.02	−0.29	−0.35	−0.70*	−0.24	−0.83*
Mg	0.56*	0.21	0.68*	0.65*	0.60	0.52*	0.76*
Zn	0.20	−0.05	0.25	0.15	0.67*	0.16	0.62*
age	−0.03	0.26	−0.27	−0.22	0.79*	0.00	0.12
Pfirrmann	−0.06	−0.13	−0.04	−0.09	0.00	0.12	−0.42

*Statistically significant

**Fig. 1:**
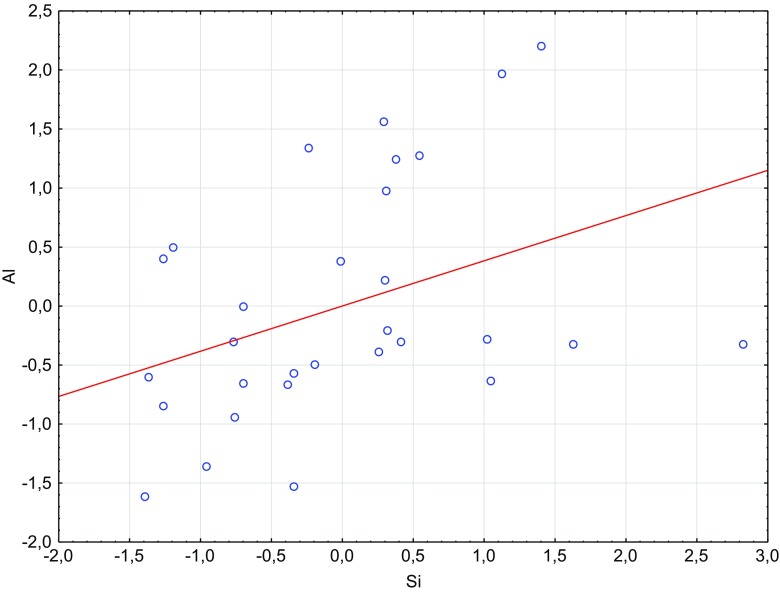
Scatterplot of standardized Si against standardized Al

**Fig. 2:**
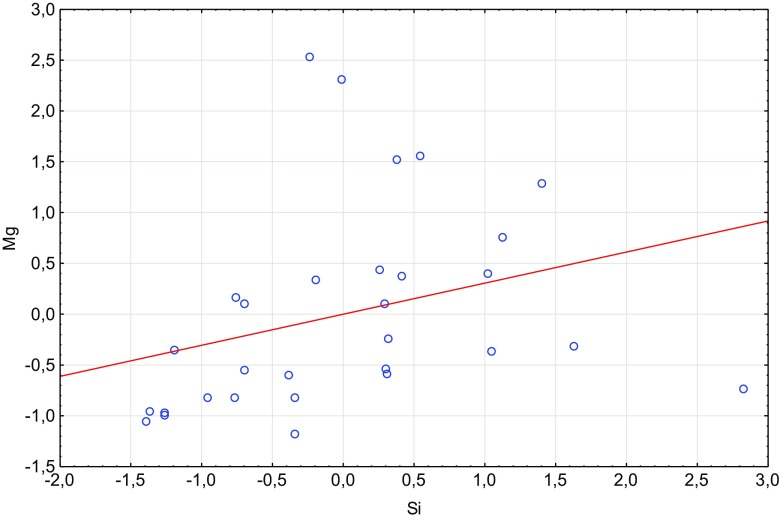
Scatterplot of standardized Si against standardized Mg

**Fig. 3:**
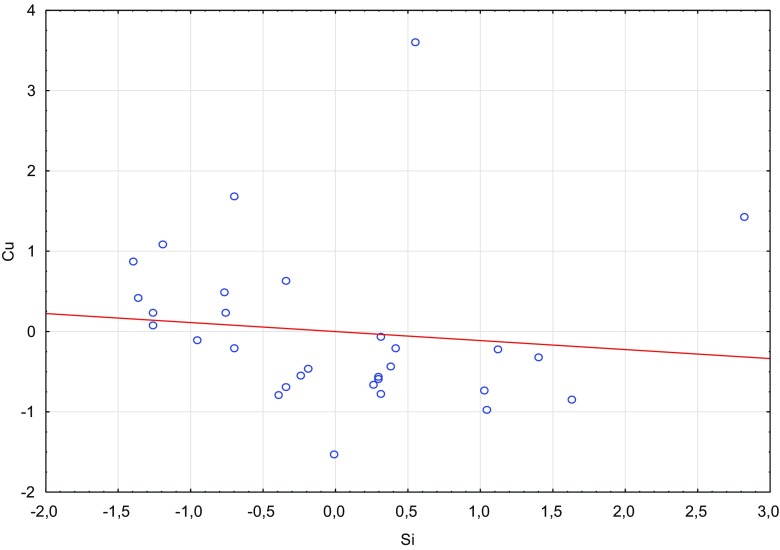
Scatterplot of standardized Si against standardized Cu

**Fig. 4:**
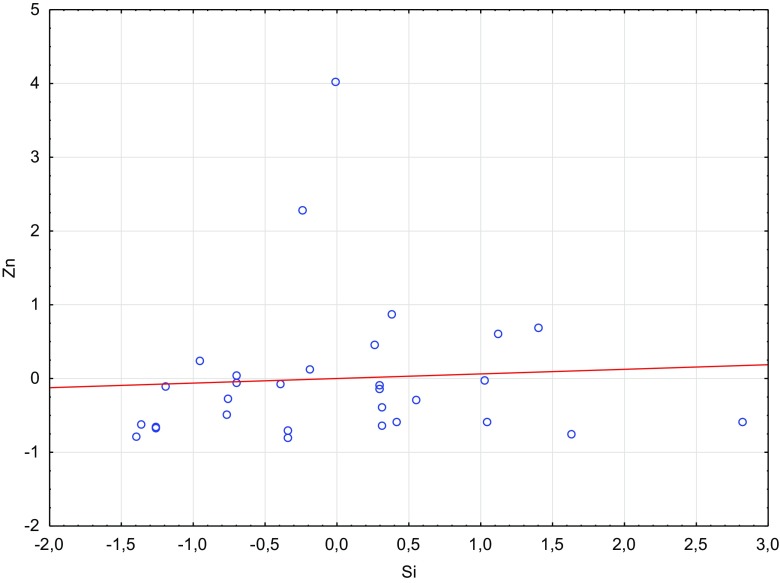
Scatterplot of standardized Si against standardized Zn

In order to define correlations between silicon and the other metals determined in intervertebral disc samples, the similarity analysis was performed. The distribution analysis for silicon showed similarities between concentrations of both Al and Mg (Figs. 1 and 2), while the distribution patterns between the following elements Si-Cu and Si-Zn confirmed no similarities between them (Figs. 3 and 4).

**Fig. 5:**
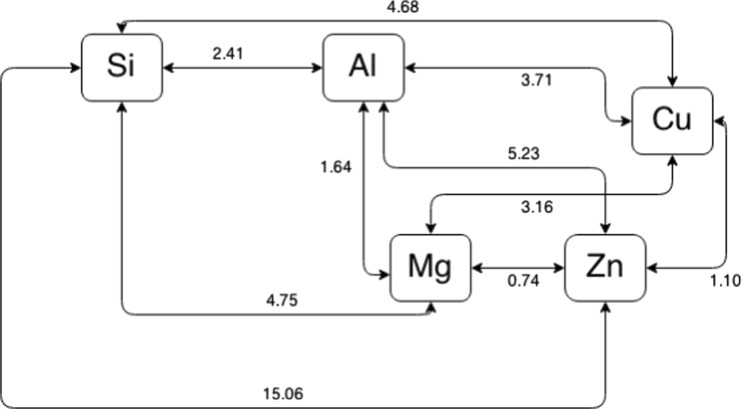
Smallest-distance graph

Based on tangent distance (*d* ^T^) matrix, the smallest distances graph was performed (Fig. 5) and two main groups were distinguished. The first group included Mg, Zn, and Cu, while the second group included Si and Al. Although the distance between Mg and Al was smaller than that between Al and Si (1.64 and 2.41, respectively), the distances between Al and the other analyzed elements were relatively high. The graph (Fig. 5) presents lack of similarities between Si and other metabolically related elements, i.e., Mg, Zn, and Cu.

## Discussion

The majority of studies dealing with silicon in human tissues were performed in patients with breast implants, due to growing popularity of the cosmetic surgery. The research of McConnell et al. (1997) showed that the silicon level in parenchymal tissue of natural (not augmented) breasts was 64 μg g^−1^, whereas Peters et al. (1996) determined much lower concentrations of silicon in 16 breast tissue samples, from 0.046 to 0.742 μg g^−1^of dry weight, with the mean value of 0.0927 μg g^−1^. Other studies showed mean Si concentrations of 12–27 μg g^−1^ for human tissues, including the brain, and gave total body burden values of several grams (D’Haese et al. 1995). In this study, we generally determined much higher concentrations of Si in human tissues (IVD) than other authors, except for McConnell et al. (1997). The concentrations of Si measured in degenerated intervertebral disc tissues in humans were within the range of 5.37–12.8 μg g^−1^. This justifies to consider silicon as an element that is somewhere between the major and minor dietary elements. The potential role of the silicon in the intervertebral disc can be derived from previous studies on animals and humans. It has been confirmed that silicon supplementation can promote new bone formation and regeneration, both in fracture healing and osteoporosis (Jugdaohsingh et al. 2004; Nielsen 2014). The human cohort studies suggested that higher silicon intake is associated with higher bone mineral density (BMD) and with higher bone remodeling marker level (Jugdaohsingh et al. 2004; MacDonald et al. 2012). Silicon deficiency may cause decreased concentrations of Ca, Mg, Cu, K, and Zn in bones (Seaborn and Nielsen 2002). We confirmed this fact in our distance analysis. Also, silicon can influence the absorption, retention, and behavior of mineral elements (Al, Mg, and Cu) (Nielsen 2014). In this study, we statistically confirmed strong relationship between Si and Al and between Si and Mg in human spinal tissues. It is worth noticing that our research, for the first time, confirmed the similarity between Si and Al for human intervertebral disc tissue. Our observations are in compliance with those which show a strong relationship of silicon and aluminum, both in humans and in biota (Exley 2009a, b; Davenward et al. 2013). Birchall et al. (1996) suggested that deficiency of silicon and its influence on collagen and osteogenesis may be due to low copper utilization. Based on the results of the present study, the similarities between Si and Cu and also between Si and Zn cannot be proved. The relationship between Si content and age was not statistically confirmed. However, higher Si concentrations were determined in younger female patients (age range 28–34 years) as compared to older ones (42–64 years). Jugdaohsingh et al. (2013) also found higher Si concentration in the serum of the neonates as compared to the adults. The study of silicon content in the connective tissues from human aorta showed that Si decreased with age.

## Conclusions

Although many studies have been devoted to the supplementation of humans with silicon and to determining the effect of Si on the human body, especially on bones, there are no studies that deal with silicon content of human spinal tissues (Davenward et al. 2013; Pruksa et al. 2014; Jugdaohsingh et al. 2013; Li et al. 2010). This can be confirmed by the review of Rodella et al. (2014) which proves beneficial effect of Si on human bones but contains no information on the studies of Si content in human bone tissues nor in spine tissues. Our study, for the first time, presents the results of silicon analysis in the human intervertebral disc. We confirmed strong correlation between Si and Al in the analyzed tissues. Taking into account the general statement that silicon limits the toxicity of aluminum (Birchall et al. 1989; Van Landeghem et al. 1998; Parry et al. 1998; Exley 2009a), our results can be fundamental information which will help to recognize the mechanism of interaction between Si and Al in the human body
